# The effects of virtual reality training on cognitive function in older adults: a systematic review and meta-analysis

**DOI:** 10.3389/fpubh.2026.1823497

**Published:** 2026-05-20

**Authors:** Yeqin Lian, Xingyi Li, Changzhou Chen

**Affiliations:** 1 School of Physical Education, Shanghai University of Sport, Shanghai, China; 2 Department of Sport and Health, Shinhan University, Dongducheon, Gyeonggi, Republic of Korea

**Keywords:** cognition, older adults, executive function, meta-analysis, virtual reality

## Abstract

**Objectives:**

Virtual reality (VR) has emerged as a promising non-pharmacological approach for supporting cognitive health in older adults. However, the overall magnitude of its effects and the extent to which outcomes vary by cognitive status, intervention duration, and immersion level remain uncertain.

**Methods:**

Randomized controlled trials (RCTs) were searched in Web of Science, PubMed, Scopus, Embase, Cochrane Library, and Wanfang Database from database inception to September 24, 2025, without language restrictions. Eligible studies included older adults receiving VR-based training compared with routine rehabilitation, conventional training, no intervention, or non-VR controls. Outcomes were classified as global cognition, TMT-based executive/attention-related performance, and functional mobility. Risk of bias was assessed using the Cochrane RoB 2 tool. Random-effects meta-analyses were conducted using standardized mean differences (SMDs) with 95% confidence intervals (CIs). Subgroup analyses were interpreted as exploratory.

**Results:**

Ten RCTs involving 749 participants were included. VR-based training significantly improved global cognition (SMD = 0.49, 95% CI: 0.25–0.73, *p* < 0.0001), with moderate heterogeneity (*I*^2^ = 50%). Functional mobility, analyzed as a secondary outcome, also improved (SMD = 0.39, 95% CI: 0.05–0.73, *p* = 0.02; I^2^ = 56%). Only one trial reported TMT-based executive/attention-related outcomes using TMT-A and TMT-B. Because these outcomes were derived from the same study population, they were not interpreted as evidence from independent studies; the findings suggested a favorable direction of effect but were considered preliminary. Exploratory subgroup analyses suggested possible variation by cognitive status, intervention duration, and immersion level, but several comparisons were based on small numbers of studies.

**Conclusion:**

VR-based training may improve global cognition in older adults and may also benefit functional mobility. Evidence for TMT-based executive/attention-related performance remains preliminary. Current evidence is insufficient to support firm conclusions regarding optimal intervention duration, preferred immersion level, or subgroup superiority. Larger, well-designed RCTs with standardized outcome classification and longer follow-up are needed.

**Systematic review registration:**

https://www.crd.york.ac.uk/PROSPERO/view/CRD420251208123, Identifier: CRD420251208123.

## Introduction

Population ageing is accelerating worldwide. According to the World Health Organization, the proportion of people aged 60 years and older is expected to rise substantially over the coming decades, and China is also experiencing a rapid expansion of its older population (1). Alongside ageing, declines in processing speed, memory, and executive function become increasingly common, even among otherwise healthy older adults, and may adversely affect independence and quality of life (2). A proportion of older adults experience cognitive decline beyond the normal ageing range and develop mild cognitive impairment (MCI), also referred to as mild neurocognitive disorder (3–6). MCI is highly prevalent in later life; pooled evidence suggests a global prevalence of approximately 23.7% among adults aged 60 years and older (7),and community-based data from China similarly indicate a substantial burden (8). MCI is clinically important because it is associated with a markedly increased risk of progression to dementia (3, 9), making it a critical window for prevention and early intervention (10). Given the absence of specific pharmacological treatments capable of reversing or halting early cognitive decline (9), effective non-pharmacological strategies for maintaining or improving cognitive function in older adults remain a major public health priority.

Virtual reality (VR) has emerged as a promising interactive approach in the field of cognitive enhancement and rehabilitation (11). Compared with traditional paper-and-pencil or conventional computer-based cognitive training, VR can deliver multisensory, task-oriented, and ecologically relevant experiences that may improve engagement and adherence among older adults (11, 12). Existing studies suggest that VR-based training may support cognitive performance through repeated practice, real-time feedback, and the simultaneous stimulation of cognitive and sensory systems, although these mechanistic explanations remain inferential rather than definitive (3, 13, 14). Accordingly, VR has increasingly been considered a potentially useful adjunctive intervention for supporting cognitive health in older populations.

Importantly, VR is not a single intervention format. It is commonly classified as non-immersive, semi-immersive, or immersive (15, 16). Non-immersive VR generally involves screen-based interaction through devices such as desktop computers, televisions, keyboards, mice, or motion-sensing systems, and is often used in serious games or video game-based training programs (17). By contrast, immersive VR may provide a stronger sense of presence and richer interactive stimulation, but it may also impose greater adaptation demands and may cause discomfort, such as cybersickness, particularly in older users (18). As a result, whether greater immersion necessarily leads to superior cognitive outcomes remains uncertain (12). Previous evidence syntheses have reported inconsistent findings regarding the comparative effects of immersive, semi-immersive, and non-immersive VR on global cognition and executive function in older adults with MCI (15, 19). In addition, a dedicated meta-analysis of fully immersive VR reported significant benefits for global cognition, executive function, and attention, whereas the effect on memory was less clear (20). Together, these findings suggest that immersion level may influence intervention effects, but the direction and magnitude of this influence have not yet been established consistently.

At the trial level, randomized controlled studies have reported encouraging findings in both cognitively healthy older adults and older adults with MCI. For example, Gamito et al. found that VR-based cognitive stimulation improved several neuropsychological outcomes in community-dwelling healthy older adults (21). whereas Thapa et al. reported that an 8-week VR intervention improved executive function and related outcomes in older adults with MCI (22). Positive results have also been reported for VR-based cognitive-motor dual-task interventions, and meta-analytic evidence suggests favorable effects on global cognition in older adults with MCI (23). Moreover, VR has also been explored in dementia-related care, including reminiscence-based applications (18). Nevertheless, the existing evidence base remains limited by small sample sizes, short follow-up periods, substantial heterogeneity in intervention content and dosage, and inconsistent comparisons across studies (12, 14). Previous reviews have also often focused on specific populations or have combined conceptually different intervention types, which makes it difficult to determine whether effects differ according to baseline cognitive status or immersion level (12, 19).

Therefore, a comprehensive and methodologically rigorous synthesis is needed. The present systematic review and meta-analysis aimed to evaluate the effects of VR-based interventions on cognitive function in older adults and to explore whether effect estimates differed according to cognitive status, intervention duration, and immersion level. In the revised analytical framework, cognitive outcomes were considered separately from functional mobility outcomes in order to improve conceptual clarity.

### Study objectives

To improve conceptual precision, the present review distinguished broad cognitive screening outcomes from domain-specific neuropsychological outcomes and analyzed functional mobility separately from cognition. Specifically, this review aimed to: (1) estimate the overall effect of VR-based interventions on selected outcomes in older adults; (2) examine whether pooled effects differed according to baseline cognitive status; and (3) explore whether intervention duration and immersion level were associated with variability in effect estimates. Given the expected heterogeneity of interventions and outcome measures, subgroup findings were interpreted as exploratory rather than confirmatory.

## Methods

### Research methodology

This systematic review and meta-analysis followed the recommendations outlined in the Cochrane Manual of Interventional Systematic Reviews (6.5), was reported strictly in accordance with the PRISMA Reporting Guidelines (PRISMA 2020 Statement), and was registered in the International Registry of Prospective Systematic Reviews on September 10, 2025 (registration number CRD420251208123) (24, 25).

### Search strategy

Searches were conducted in Wanfang Database, Scopus, PubMed, Cochrane Library, Embase, and Web of Science, from the database inception date to September 24, 2025, No language restrictions were applied at either the search or screening stage. Records were screened according to the predefined eligibility criteria regardless of publication language. When potentially relevant non-English records were identified, they were assessed on the basis of translated titles/abstracts or full texts where necessary. Articles were excluded only because they failed to meet the eligibility criteria or did not provide extractable outcome data, not because of publication language. The search strategy was designed based on the Population, Intervention, and Outcome (PIO) framework, using a combination of MeSH keywords and free terms to construct the search query. Two researchers independently screened the literature, reviewing titles and abstracts to exclude obviously irrelevant studies, strictly adhering to inclusion and exclusion criteria to determine if the literature met the inclusion requirements. In case of disagreement, the issues were discussed and resolved first; if necessary, a third reviewer was involved to reach a consensus before the study was included.

The PubMed search strategy was as follows:

((“Aged”[Mesh] OR aged[Title/Abstract] OR elderly[Title/Abstract] OR older adult*[Title/Abstract] OR senior*[Title/Abstract] OR geriatric*[Title/Abstract])AND(“Virtual Reality”[Mesh] OR “virtual reality”[Title/Abstract] OR VR[Title/Abstract] OR immersive[Title/Abstract] OR non-immersive[Title/Abstract] OR semi-immersive[Title/Abstract] OR “head-mounted display”[Title/Abstract] OR HMD[Title/Abstract] OR exergame*[Title/Abstract] OR “active video game*”[Title/Abstract] OR videogame*[Title/Abstract] OR “video game*”[Title/Abstract] OR “serious gam*”[Title/Abstract] OR “computerized training”[Title/Abstract] OR “interactive training”[Title/Abstract])AND(“Cognition”[Mesh] OR cognition[Title/Abstract] OR cognitive[Title/Abstract] OR “cognitive function”[Title/Abstract] OR “mild cognitive impairment”[Title/Abstract] OR MCI[Title/Abstract] OR memory[Title/Abstract] OR attention[Title/Abstract] OR “executive function”[Title/Abstract] OR MoCA[Title/Abstract] OR MMSE[Title/Abstract] OR “Trail Making Test”[Title/Abstract] OR TMT[Title/Abstract])AND(randomized controlled trial[Publication Type] OR randomized[Title/Abstract] OR randomized[Title/Abstract] OR randomly[Title/Abstract] OR trial[Title/Abstract] OR RCT[Title/Abstract]))

### Inclusion and exclusion criteria

Inclusion criteria: (1) Studies using a randomized controlled trial (RCT) design; (2) participants were older adults aged 60 years or above with either normal cognitive function or mild cognitive impairment (MCI); (3) the intervention group received a VR-based training program, including immersive, semi-immersive, or non-immersive VR; (4) the control group received routine care, health education, no intervention, conventional exercise, or other non-VR training; (5) the study reported at least one extractable cognitive or functional outcome measure, such as MoCA, MMSE, TMT, or TUG.

Exclusion criteria: (1) participants had a diagnosed neurodegenerative dementia disorder, including Alzheimer’s disease at any stage (including mild AD), or other severe neurological disease that substantially limited training participation or outcome assessment; (2) participants had severe cardiovascular, cerebrovascular, or other disorders that severely restricted motor performance; (3) VR was combined with other interventions that could not be analytically separated from the effect of VR itself; (4) the article was a duplicate publication or did not provide extractable outcome data. To maintain conceptual consistency, the present review focused on cognitively healthy older adults and individuals with MCI; therefore, studies enrolling participants with diagnosed Alzheimer’s disease, including mild AD, were not included.

### Definition of VR immersion level

VR interventions were classified as non-immersive, semi-immersive, or immersive according to the level of sensory enclosure and interaction. Non-immersive VR referred to screen-based or monitor-based systems in which participants interacted with a virtual environment while remaining fully aware of the surrounding real-world setting. Immersive VR referred to interventions delivered through head-mounted displays or comparable systems that substantially occluded the external environment and created a strong sense of presence within a fully enclosed virtual space. Semi-immersive VR was operationally defined as an intermediate format in which participants interacted with a virtual environment through simulator-based, projection-based, or large-screen interactive systems that provided enhanced environmental engagement and task-specific interaction, but did not fully occlude the real-world environment or rely on head-mounted full immersion. To enhance reproducibility, studies were classified according to the primary delivery hardware and degree of environmental occlusion rather than the terminology used by the original authors alone. Head-mounted systems that substantially blocked the external environment were classified as immersive; large-screen, projection-based, or simulator-style systems that enhanced virtual engagement without full enclosure were classified as semi-immersive; and standard monitor-based systems were classified as non-immersive.

### Data extraction

#### Outcome classification and data extraction

To improve conceptual clarity, outcomes were classified before synthesis into three domains: (1) global cognition, including broad cognitive screening measures such as the Montreal Cognitive Assessment (MoCA) and Mini-Mental State Examination (MMSE); (2) TMT-based executive/attention-related performance, including Trail Making Test Part A (TMT-A) and Trail Making Test Part B (TMT-B); and (3) functional mobility, represented by the Timed Up and Go test (TUG). TUG was analyzed separately as a functional mobility outcome and was not grouped within the cognitive outcome framework. Because TMT-A and TMT-B assess related but non-identical constructs and were derived from the same study population in the included evidence, these outcomes were not interpreted as independent studies when evaluating TMT-based executive/attention-related findings.

Data extraction tables were pre-designed, specifying the main extraction contents, including: ① basic information of the literature (first author, publication year); ② characteristics of the research subjects; ③ sample size; ④ intervention protocol; ⑤ control measures; ⑥ outcome indicators (Moca, MMSE, TMT-A, TMT-B, TUG). All data were extracted independently by two researchers, and any inconsistencies were resolved by a third researcher. The merged research data were in the form of mean ± standard deviation (Mean ± SD). The difference in means and standard deviation before and after the intervention were calculated using the Evidence-Based Medicine Assistant Indicator Calculation and Transformation Tool. The formula for calculating the difference in means is: *M*_change_ = M_final_- M_baseline_, *M*_change_is the difference between the means before and after the experiment.; *M*_final_ is the post-trial mean.;*M*_baseline_ is the pre-experiment mean. The standard deviation change formula is: SD_change_ = $\sqrt{{\mathrm{SD}}_{\text{baseline}}^{2}+{\mathrm{SD}}_{\text{final}}^{2}-(2\times \text{Corr}\times {\mathrm{SD}}_{\text{baseline}}\times {\mathrm{SD}}_{\text{final}})}$ , where SD_change_ is the difference in standard deviation between pre- and post-intervention; ${\mathrm{SD}}_{\text{baseline}}$ is the pre-intervention standard deviation, and ${\mathrm{SD}}_{\text{final}}$ is the post-intervention standard deviation. Corr denotes the correlation coefficient, which is set at 0.8 (26, 27). For standard deviations requiring conversion, the Cochrane standard deviation conversion tool is employed, based on confidence intervals and sample size., When the sample size ≤60, calculations utilize the t-distribution, with the conversion formula: SD = $\frac{\sqrt{N}\times ({\mathrm{CI}}_{\text{upper}}-{\mathrm{CI}}_{\text{lower}})}{2\times t_{\alpha /2,\mathrm{df}}}$ , α = 0.05, corresponds to a 95% CI, and degrees of freedom df = N − 1; When the sample size >60, the formula simplifies to SD = $\frac{\sqrt{N}\times ({\mathrm{CI}}_{\text{upper}}-{\mathrm{CI}}_{\text{lower}})}{3.92}$ .

### Research bias risk assessment

The methodological quality of the included studies was assessed using the Cochrane Risk of Bias 2 (RoB 2) tool for randomized controlled trials. RoB 2 evaluates bias across five domains: (1) bias arising from the randomization process; (2) bias due to deviations from intended interventions; (3) bias due to missing outcome data; (4) bias in measurement of the outcome; and (5) bias in selection of the reported result. Each domain was judged as “low risk of bias,” “some concerns,” or “high risk of bias,” and an overall risk-of-bias judgment was assigned for each study. During assessment, two reviewers independently read the full texts and made item-by-item judgements. Disagreements were resolved through discussion, with a third reviewer invited for arbitration when necessary.

### Statistical methods

All statistical analyses were conducted using Review Manager 5.3 and Stata 18. Because conceptually similar outcomes were measured using different instruments across studies, pooled effect sizes were expressed as standardized mean differences (SMDs) with 95% confidence intervals (CIs) (28). The primary cognitive outcome was global cognition. TMT-based executive/attention-related performance was analyzed separately because the available TMT-A and TMT-B data were derived from a single trial. Functional mobility, assessed using TUG, was analyzed separately as a secondary outcome. For all pooled analyses, random-effects models were used *a priori* to account for expected clinical and methodological heterogeneity across studies. Statistical heterogeneity was assessed using Cochran’s Q test and the *I*^2^ statistic. A *p*-value <0.10 for the *Q* test or an *I*^2^ value >50% was considered indicative of substantial heterogeneity. Sensitivity analyses were performed using sequential study exclusion to examine the robustness of the pooled estimates. Prespecified subgroup analyses were conducted according to participant cognitive status, intervention duration, and VR immersion level. Given the limited number of studies contributing to several subgroup comparisons, these analyses were considered exploratory and were interpreted cautiously. Publication bias was explored using funnel plots, Begg’s test, and Egger’s test where feasible. However, because fewer than 10 studies were available for each pooled outcome, these assessments were considered underpowered and exploratory rather than confirmatory. Statistical significance was set at *α* = 0.05.

## Results

### Study selection

**Figure 1:**
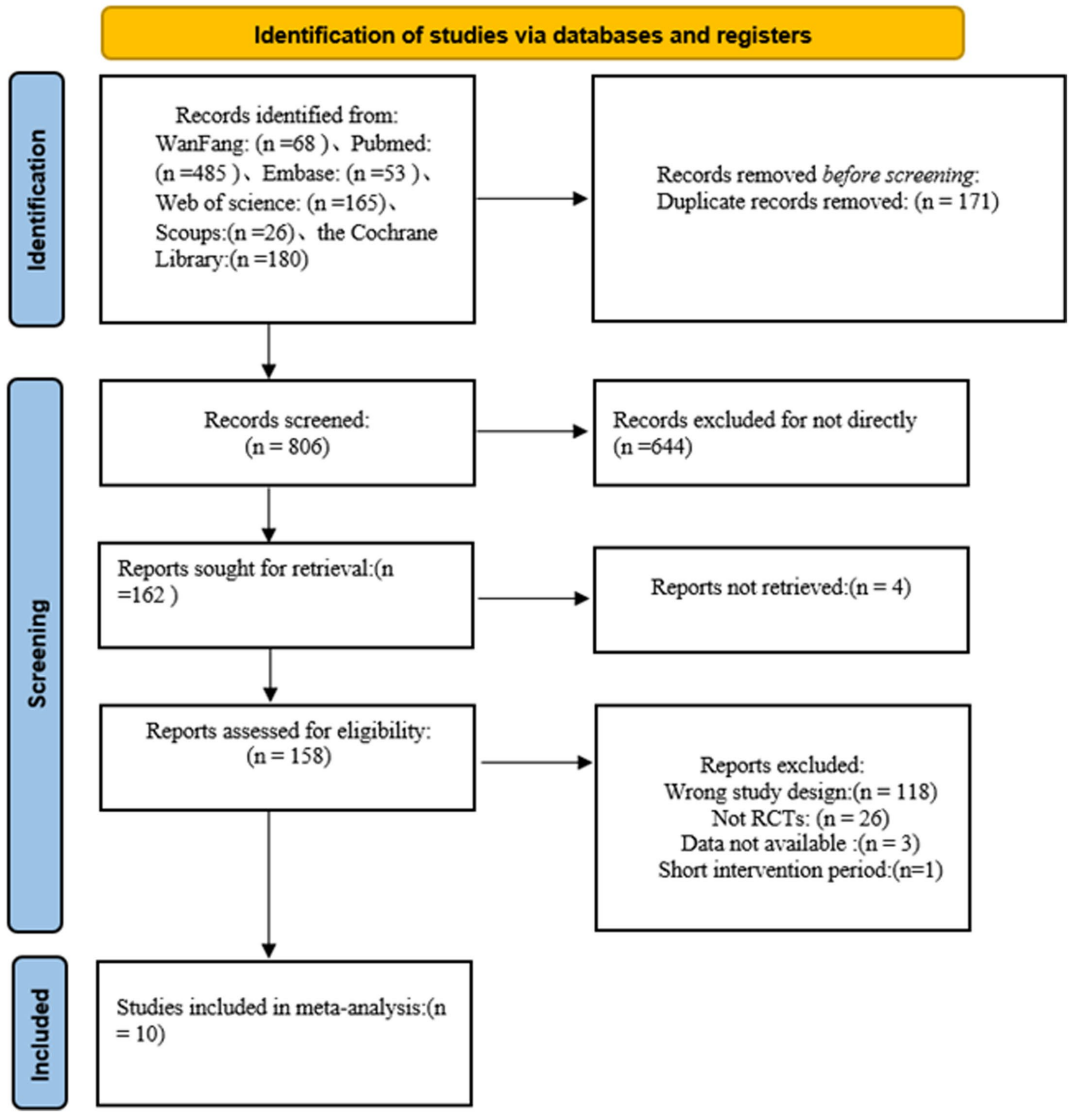
Flow diagram of literature screening and inclusion.

A total of 977 records were identified through database searching. After duplicate removal and screening of titles and abstracts, potentially eligible full-text articles were assessed according to the predefined inclusion and exclusion criteria. Ten randomized controlled trials met the final eligibility criteria and were included in the meta-analysis, comprising 749 participants. No language restrictions were applied during either the search or screening process. Although both Chinese and international databases were searched, all studies that ultimately met the eligibility criteria and provided extractable data were published in English. No study was excluded solely because of publication language. The study selection process is shown in Figure 1.

### Study characteristics

A total of 10 randomized controlled trials were included in this review, involving 749 participants aged 60 years and older. The included studies enrolled both cognitively healthy older adults and older adults with mild cognitive impairment (MCI). The intervention groups received VR-based training delivered in immersive, semi-immersive, or non-immersive formats, whereas control groups received routine care, health education, conventional exercise, non-cognitive leisure activities, or no intervention.

The intervention content varied considerably across studies and included gamified cognitive training, motor-cognitive dual-task training, virtual rowing or kayaking tasks, driving simulation, balance and gait training, and immersive cognitive training. Training frequency generally ranged from one to three sessions per week, and intervention duration ranged from 6 to 8 weeks. Session duration typically ranged from 20 to 60 min when reported. This variation in participant characteristics, intervention content, comparator conditions, and training dosage suggests the presence of substantial clinical heterogeneity across the included studies. Detailed characteristics of the included studies are presented in Table 1.

**Table 1:** Characteristics of the Included Studies.

Included in the study	Author’s country	Average age	Number of people (T/C)	Intervention measures T C		Immersion category	Demographic characteristics	Intervention duration	Intervention frequency	Outcome indicator
Ngee Masara Thapa 2020 (22)	China	>65	33/33	Fully Immersive VR Training	Health Education	Immersive VR training	MCI older adults	Eight weeks	Three times	①②③
Jun yuck Park 2016 (29)	Korea	72.97 ± 2.98	36/36	VR-Kayak	Regular exercise	Non-immersive VR training	Normal older adults	Six weeks	Two times	⑤
Hadi Nobari 2021 (30)	Iran	71.5	20/20	Virtual Reality Driving Training	Regular exercise	Semi-immersive VR training	Normal older adults	Six weeks	Three times	①④
Simona Mrakic-Sposta 2018 (31)	Italy	72.0 ± 5.15	5/5	Non-immersive VR combined with treadmill platform training	non-intervention	Non-immersive VR training	MCI older adults	Six weeks	Three times	①
Rick Yiu Cho Kwan 2021 (32)	Switzerland	74.2 ± 5.5	9/8	VR motor-cognitive exercise	Regular exercise	Semi-immersive VR training	MCI older adults	Six weeks	Three times	④⑤
Rick Yiu Cho Kwan 2024 (33)	HongKong	≥60	146/147	VR Motor-Cognitive Training	Regular exercise	Non-immersive VR training	Normal older adults	Eight weeks	Two times	④⑤
Tom Delbroek 2017 (34)	Belgium	87.2 ± 5.96	10/10	Dual-task VR + BioRescue	Regular exercise	Non-immersive VR training	MCI older adults	Six weeks	Two times	④⑤
Chuang, I-Ching 2025 (35)	China	≥60	68/69	Immersive VR training	non-cognitive leisure activities	Immersive VR training	Normal older adults	Eight weeks	Two times	④⑤
Wonjae Choi and Seungwon Lee 2019 (36)	Korea	≥65	30/30	Virtual Kayaking Training	Home exercise	Non-immersive VR training	Normal older adults	Six weeks	Two times	⑤
Jorge Buele 2024 (37)	Spain	65–86	17/17	Group sports training + VR cognitive training	physical training	Immersive VR training	MCI older adults	Six weeks	Two times	⑤

① MMSE; ② TMT-A; ③ TMT-B; ④ TUG; ⑤ MOCA.

### Bias risk assessment

**Figure 2:**
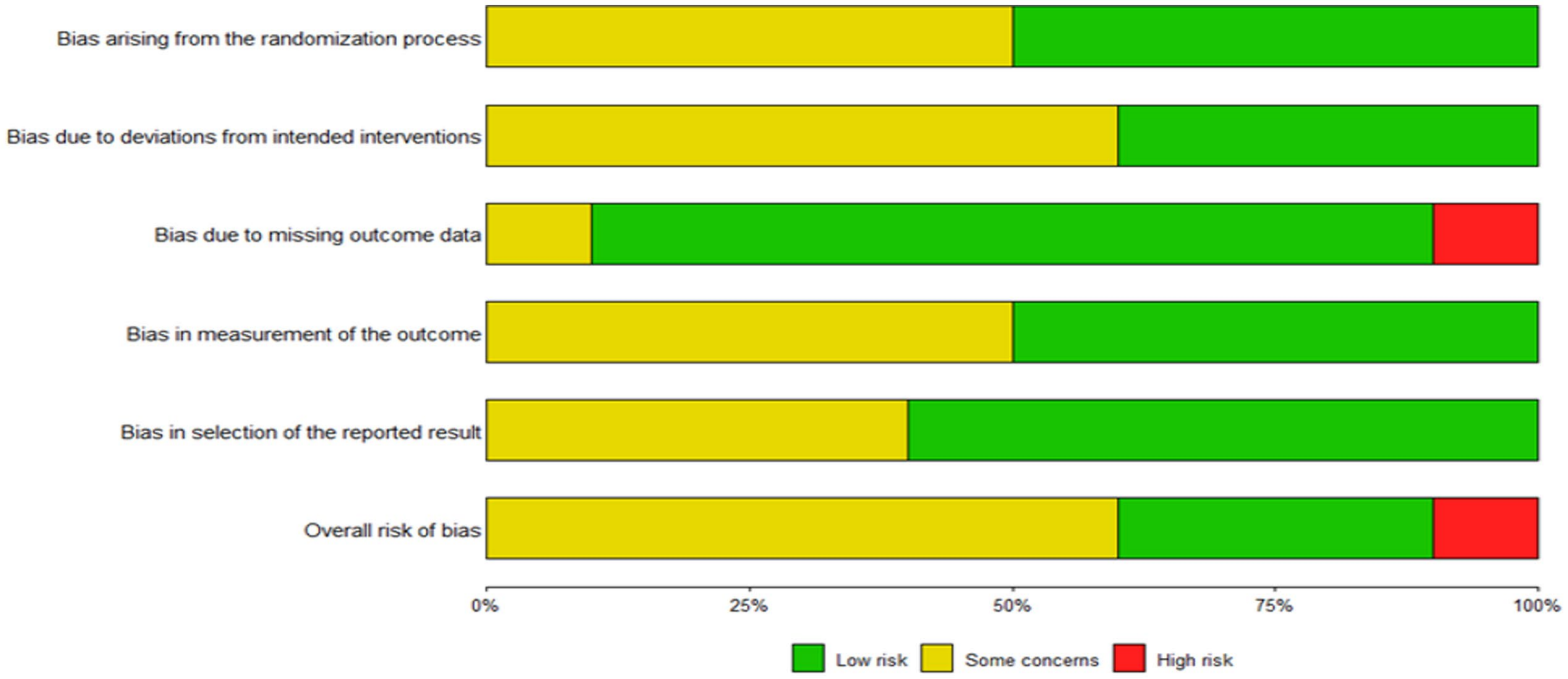
Risk of bias bar chart.

**Figure 3:**
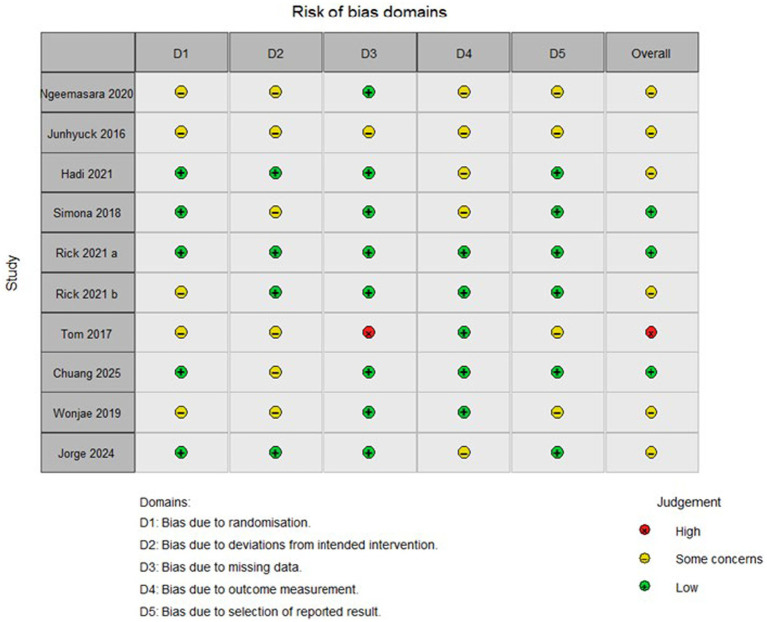
Summary of the risk of bias.

Risk of bias was assessed using the Cochrane Risk of Bias 2 (RoB 2) tool (Figures 2, 3). Across the 10 included studies, 3 studies were judged as low risk of bias overall, 6 were judged as having some concerns, and 1 were judged as high risk of bias. The most frequent concerns arose from the randomization process and from deviations from intended interventions, largely because several studies provided limited detail on allocation procedures and blinding was inherently difficult in behavioral VR interventions. Concerns related to missing outcome data, outcome measurement, and selective reporting were less frequent but were present in some trials. Overall, the certainty of the evidence was limited by variability in methodological rigor across the included studies.

### Meta-analysis and sensitivity analysis

**Figure 4:**
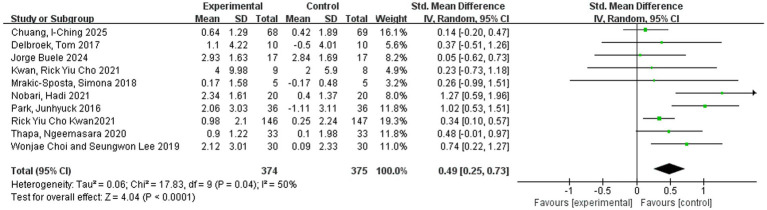
The impact of virtual reality training on the overall cognitive function of older adults.

**Figure 5:**
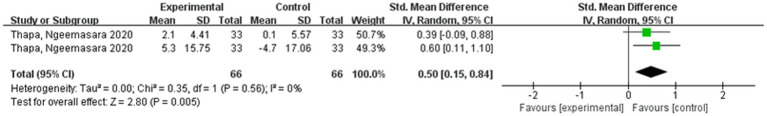
The impact of virtual reality training on TMT-based executive/attention-related performance in older adults.

**Figure 6:**
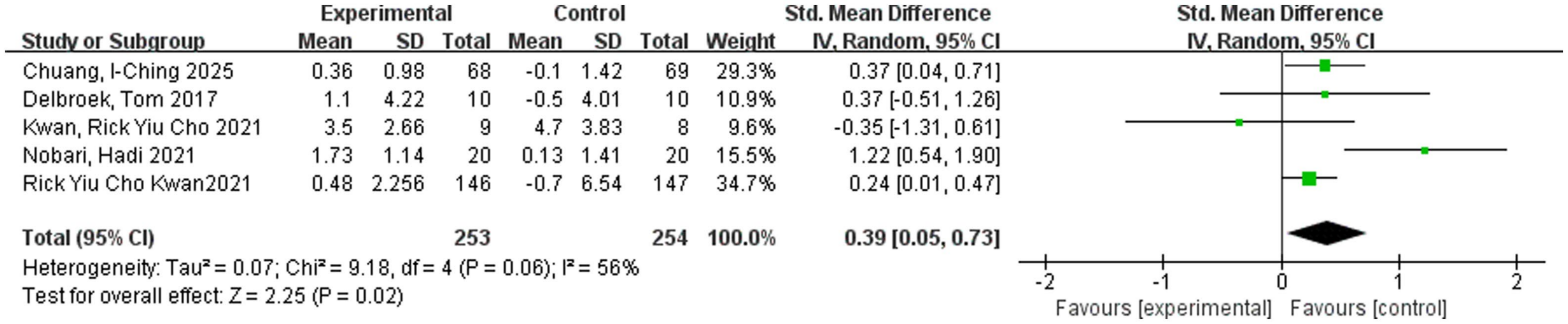
The impact of virtual reality training on functional mobility in older adults.

**Figure 7:**
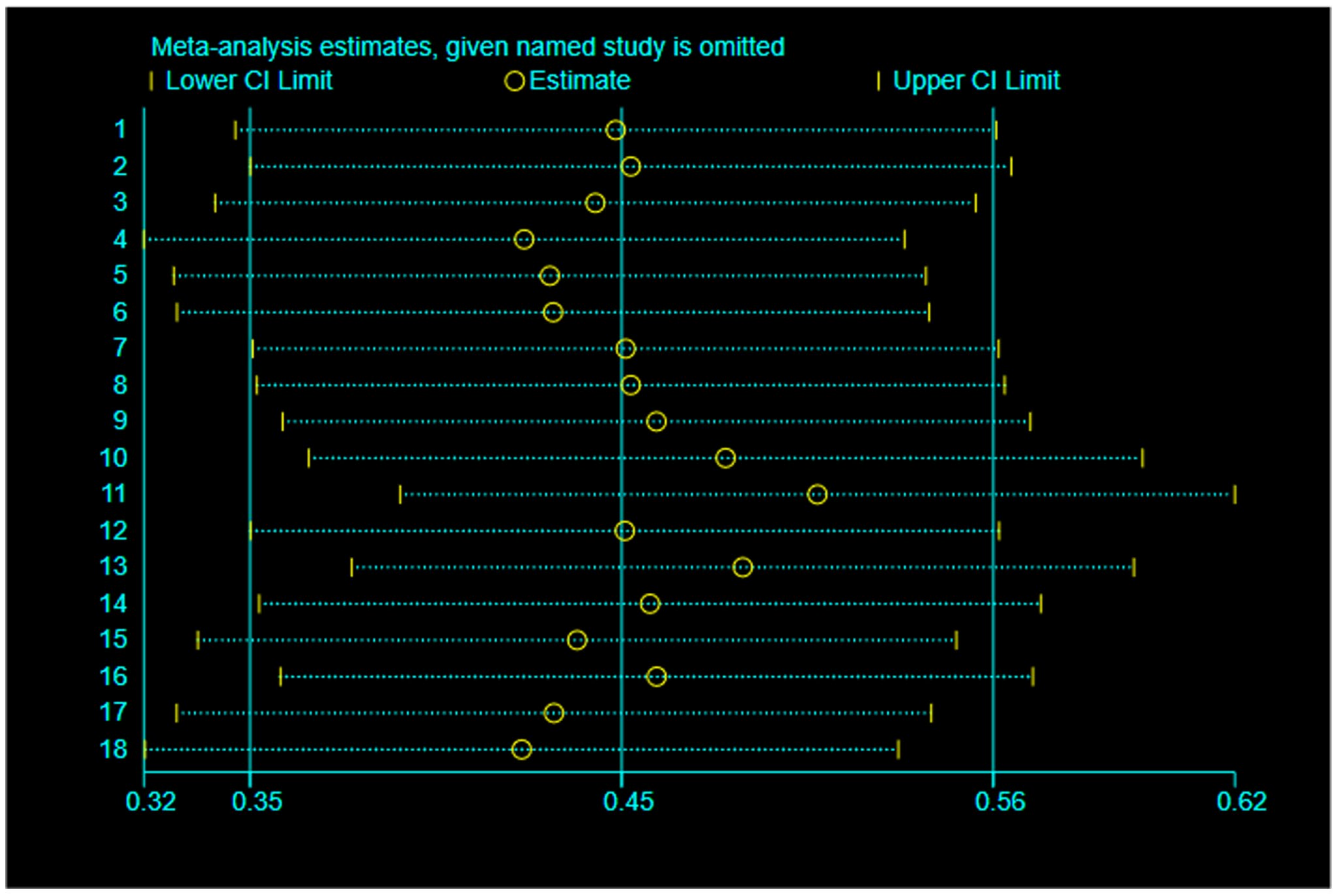
Sensitivity analysis.

The pooled effect sizes are presented in Figures 4–6. In the revised analytical framework, cognitive outcomes and functional mobility outcomes were analyzed separately. For global cognition, pooled analysis showed that VR-based interventions significantly improved cognitive performance in older adults (SMD = 0.49, 95% CI: 0.25–0.73, *p* < 0.0001), with moderate heterogeneity (*I*^2^ = 50%). Although the combined TMT-based estimate was SMD = 0.50 (95% CI: 0.15–0.84), but this value should be interpreted cautiously because it was based on two related outcomes from one trial rather than on multiple independent trials. Functional mobility, analyzed separately using TUG, showed [SMD = 0.39, 95% CI: 0.05–0.73, *p* = 0.02], with heterogeneity of [*I*^2^ = 56%]. These findings indicate that VR-based interventions may provide benefits across selected domains, although effect sizes and precision varied across outcomes. Figure 7 presents the results of a leave-one-out sensitivity analysis, which sequentially excluded individual studies to assess the robustness of the overall effect. As illustrated, removing each study individually maintained the pooled effect size estimates within the range of 0.31 to 0.56, with none falling outside the confidence interval of the overall effect size (approximately 0.41). No single study caused a reversal in effect direction or significant bias, indicating that the overall findings are not dependent on any particular study. These results suggest that this meta-analysis possesses good robustness and that the combined effect is highly stable.

### Publication bias

**Figure 8:**
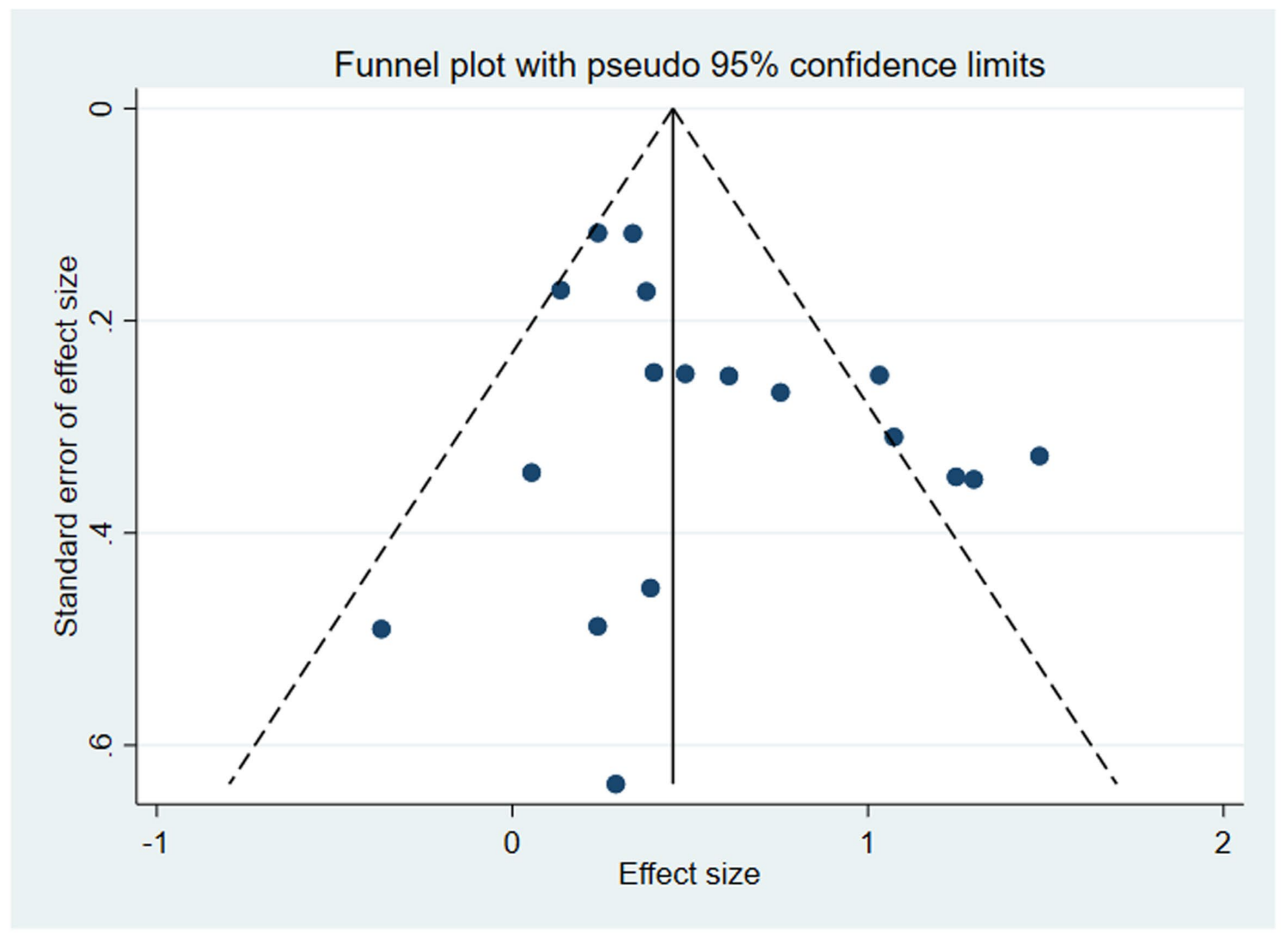
Funnel diagram.

Funnel plots were used to explore possible small-study effects and publication bias (Figure 8). Because fewer than 10 studies contributed to each pooled outcome, visual inspection of funnel plots and formal tests for small-study effects were considered descriptive only and were not regarded as capable of ruling out publication bias. Egger’s test results are presented in Table 2 for transparency. Owing to the limited number of studies, no results were generated for TMT-A or TMT-B separately. For MMSE, TUG, and MoCA, Egger’s test did not detect statistically significant asymmetry (MMSE: *p* = 0.675; TUG: *p* = 0.537; MoCA: *p* = 0.480). Nevertheless, the absence of statistically significant asymmetry should not be interpreted as definitive evidence for the absence of publication bias. Overall, publication bias and other small-study effects could not be ruled out. Accordingly, the absence of statistically significant asymmetry in Egger’s tests should not be interpreted as evidence that publication bias was absent. Rather, the available data were insufficient to support a reliable determination regarding small-study effects, and these analyses are reported for transparency only.

**Table 2:** Evaluation Results of Publication Bias of Egger test.

Testing metrics	Std_Eff	Coefficient	Std. err.	*t*	*P* > |t|	[95% conf. interval]
MMSE	Slope bias	0.6543	1.1696	0.56	0.675	−0.3090, 0.5136
	Bias	0.19965	3.4871	0.06	0.964	44.1075, 44.5068
TUG	Slope bias	−0.2159	0.3103	0.70	0.537	−0.7714, 1.2033
	bias	−0.6618	1.5433	0.43	0.697	−4.2495, 5.5731
MOCA	Slope bias	−0.2499	0.3214	0.78	0.480	−0.6424, 1.1422
	Bias	−0.7496	1.6009	0.47	0.664	−3.6951, 5.1942

### Subgroup analysis

The subgroup analysis groups and results are presented in Table 3. These subgroup analyses were prespecified according to participant cognitive status, intervention duration, and VR immersion level. Because several subgroup comparisons were based on only a small number of studies, all subgroup findings should be interpreted as exploratory. Subgroup analyses were not performed for TMT-based executive/attention-related performance because only one trial contributed relevant data.

**Table 3:** Subgroup analysis.

Outcome indicator		Study population		Intervention duration		Intervention methods		
		Normal elderly	MCI elderly	≤6 weeks	≥8 weeks	Immersive virtual reality training	Semi-immersive virtual reality training	Non-immersive virtual reality training
Global cognition	①	5	4	6	3		2	5
	②	75%	0%	56%	69%	0%	67%	45%
	③	0.63	0.33	0.45	0.64	0.22	0.81	0.58
	④	[0.26, 1.00]	[0.01, 0.68]	[0.09, 0.81]	[−0.04, 1.31]	[−0.04, 0.47]	[−0.21, 1.82]	[0.26, 0.91]
	⑤	0.0009	0.06	0.01	0.07	0.10	0.12	0.0005
Functional mobility	①	3	2	3	2	0	0	4
	②	72%	15%	0%	85%			67%
	③	0.49	0.04	0.29	0.47			0.40
	④	[0.08, 0.91]	[−0.67, 0.74]	[0.10, 0.47]	[−1.06, 2.01]			[−0.15, 0.96]
	⑤	0.02	0.92	0.002	0.55			0.15

Subgroup analyses were not conducted for TMT-based executive/attention-related performance because only one trial contributed relevant data. The blank spaces indicate that the sample size was insufficient to meet the minimum requirements for subgroup analysis. ① Number of studies; ② *I*^2^; ③ SMD; ④ 95%CI; ⑤ *p*-value.

#### Study population

When stratified by baseline cognitive status, positive pooled effect estimates were observed in both cognitively healthy older adults and older adults with MCI, although the magnitude and precision of effect estimates varied across outcomes. For global cognition, the pooled effect estimate was SMD = 0.63 (95% CI 0.26 to 1.00; *I*^2^ = 75%) in cognitively healthy older adults and SMD = 0.33 (95% CI 0.01 to 0.68; *I*^2^ = 0%) in older adults with MCI. For functional mobility, the pooled estimate was [SMD = 0.49, 95% CI 0.08 to 0.91; *I*^2^ = 72%] in cognitively healthy older adults and [SMD = 0.04, 95% CI –0.67 to 0.74; *I*^2^ = 15%] in older adults with MCI. Although some subgroup differences in point estimates were observed, these findings do not establish subgroup superiority.

#### Intervention duration

When stratified by intervention duration, pooled effect estimates differed between shorter interventions (≤6 weeks) and longer interventions (≥8 weeks), but the pattern was not uniform across outcomes. For global cognition, the pooled effect was SMD = 0.45 (95% CI 0.09 to 0.81; *I*^2^ = 56%) in the ≤6-week subgroup and SMD = 0.64 (95% CI − 0.04 to 1.31; *I*^2^ = 69%) in the ≥8-week subgroup. For functional mobility, the pooled estimate was [SMD = 0.29, 95% 0.10 to 0.47; *I*^2^ = 0%] in the ≤6-week subgroup and [SMD = 0.47, 95% CI –1.06 to 2.01; *I*^2^ = 85%] in the ≥8-week subgroup. These findings may indicate that intervention duration influences effect estimates, but they remain exploratory.

#### Intervention methods

Subgroup analyses according to immersion level (immersive, semi-immersive, and non-immersive VR) also showed variability across outcomes. For global cognition, the pooled effect estimate was SMD = 0.22 (95% CI − 0.04 to 0.47; *I*^2^ = 0%) for immersive VR, SMD = 0.81 (95% CI − 0.21 to 1.82; *I*^2^ = 67%) for semi-immersive VR, and SMD = 0.58 (95% CI 0.26 to 0.91; *I*^2^ = 45%) for non-immersive VR. For functional mobility, the pooled estimate was [SMD = 0.40, 95% –0.15 to 0.96; *I*^2^ = 67%] for non-immersive VR. Although effect estimates varied across immersion levels, these results should not be interpreted as evidence that one VR format is definitively superior to another.

Overall, subgroup analyses suggested that effect estimates may vary according to cognitive status, intervention duration, and immersion level. However, because several subgroup comparisons were based on small numbers of studies, confidence intervals were often wide, and formal tests for subgroup differences were not consistently available, these findings should be regarded as hypothesis-generating rather than confirmatory.

### GRADE evidence quality assessment

The GRADE evaluation results are presented in Table 4. The certainty of evidence for global cognition was rated as moderate. This outcome was downgraded mainly because of inconsistency, as moderate heterogeneity was observed across the included studies. The certainty of evidence for TMT-based executive/attention-related performance was rated as low. This rating was mainly due to serious imprecision, because the evidence was derived from a single trial with two related TMT outcomes from the same study population. Therefore, this finding should be interpreted as preliminary rather than as robust evidence from multiple independent trials. The certainty of evidence for functional mobility was rated as low. This outcome was downgraded because of serious risk of bias and inconsistency across the included studies. Although the pooled estimate suggested a favorable effect of VR-based training on functional mobility, the certainty of this evidence remains limited and should be interpreted cautiously.

**Table 4:** Evidence grade of the conclusion based on GRADE.

Outcome	No.of studies (effects)	Design	Total sample size (n)	Effect estimate (95% CI)	Risk of bias	Inconsistency	Indirectness	Imprecision	Other considerations	Quality
Global cognition	10	randomized trials	749	0.49 [0.25 to 0.73]	Not serious	Serious	Not serious	Not serious	None	Moderate
TMT-based executive/attention-related performance	1	randomized trials	66	0.5 [0.15 to 0.84]	Not serious	Not serious	Not serious	Serious	None	Low
Functional mobility	5	randomized trials	507	0.39[0.05 to 0.73]	serious	Serious	Not serious	Not serious	None	Low

## Discussion

### Meta results

This systematic review and meta-analysis synthesized evidence from 10 randomized controlled trials involving 749 participants and suggests that VR-based interventions may improve selected outcomes in older adults, particularly global cognition. Functional mobility may also improve, whereas evidence for TMT-based executive/attention-related performance remains preliminary because it was derived from a single trial. For global cognition and functional mobility, the pooled effects were in the small-to-moderate range, suggesting that VR may represent a promising adjunctive non-pharmacological strategy for supporting selected outcomes in later life. However, the TMT-based findings should be interpreted only as preliminary signals rather than as robust evidence of improvement in executive function.

The current findings are broadly consistent with previous randomized trials and evidence syntheses suggesting that VR-based training may benefit cognitive health in older adults (3, 13, 15, 19, 20, 23). At the same time, the present review extends prior work by jointly considering differences in baseline cognitive status, intervention duration, and immersion level, while also separating functional mobility outcomes from cognitive outcomes in the analytical framework. In the revised framework, TUG was analyzed as a functional mobility outcome rather than as a cognitive or reaction-time measure, which improves conceptual clarity and reduces the risk of overinterpreting mobility-related change as direct cognitive improvement.

The subgroup analyses suggested that pooled effect estimates for global cognition and functional mobility may vary according to participant characteristics, intervention duration, and immersion level. However, these subgroup findings were based on relatively small numbers of studies, confidence intervals were often wide, and formal between-subgroup differences were not consistently demonstrated. Therefore, these results should be viewed as exploratory and hypothesis-generating rather than as evidence of subgroup superiority or an optimal intervention protocol. TMT-based executive/attention-related performance was not examined in subgroup analyses because only one trial contributed relevant data, and no subgroup-level inference can be made for this outcome.

Several mechanisms may plausibly explain why VR-based interventions could benefit cognitive outcomes in older adults. VR can integrate multisensory stimulation, task engagement, real-time feedback, and repeated practice within ecologically relevant environments, which may plausibly support attention, task engagement, and adherence to training (11–13). However, because TMT-based executive/attention-related performance was informed by only one trial, the present review cannot establish a definitive effect of VR on executive function. In addition, immersive or interactive tasks may increase motivation and strengthen action–perception coupling. Nevertheless, these mechanistic interpretations should be considered provisional. The included trials were not designed to directly test neurobiological mechanisms, and the intervention content varied substantially across studies. Therefore, mechanistic explanations remain hypotheses that may help interpret the observed findings but should not be regarded as conclusions established by this review. Importantly, differences in subgroup-specific point estimates should not be interpreted as evidence of true subgroup superiority in the absence of robust between-subgroup interaction testing. The current subgroup findings are better understood as signals that may inform future hypothesis-driven trials rather than as a basis for definitive clinical recommendations.

The apparent differences across immersion levels may also be explained by a balance between stimulation intensity and tolerability. Immersive VR may provide stronger task engagement and more complex environmental interaction, which could theoretically increase cognitive task demands in some contexts (18, 20). However, the present evidence is insufficient to determine whether immersive VR produces superior effects on TMT-based executive/attention-related performance, because this outcome was informed by only one trial. At the same time, immersive systems may impose greater sensory and vestibular load and may reduce tolerance or adherence in some older adults, particularly those with lower cognitive reserve, poorer balance, or less familiarity with digital devices (3, 18). In contrast, non-immersive VR may provide lower task burden and better acceptability, potentially making it more suitable for repeated and sustained training. Even so, the current evidence remains insufficient to conclude that one immersion format is definitively superior to another across populations and outcomes.

Intervention duration may represent another important contributor to variability in effect estimates, particularly for global cognition and functional mobility. However, the existing data do not support a firm duration threshold. In particular, some longer-duration subgroup estimates were based on very few studies and showed wide confidence intervals. No conclusion can be drawn regarding the relationship between intervention duration and TMT-based executive/attention-related performance because only one trial contributed relevant data (4). However, the existing data do not support a firm duration threshold. In particular, some longer-duration subgroup estimates were based on very few studies and showed wide confidence intervals. Thus, although the results suggest that training duration may matter, they do not justify a definitive recommendation regarding the optimal intervention duration.

The heterogeneity observed across studies likely reflects the multifactorial nature of VR interventions. Methodologically, the included trials differed in participant characteristics, comparator types, immersion level, task content, session length, intervention frequency, and total training duration. Some interventions emphasized purely cognitive tasks, whereas others incorporated motor-cognitive dual-task components. This is particularly important because physical activity itself may contribute to cognitive improvement, making it difficult in some studies to isolate the independent effect of VR (23). Outcome measurement also varied substantially, further contributing to inconsistency across pooled estimates. Biologically, older adults differ in cognitive reserve, attentional capacity, sensory tolerance, and adaptability to technology, all of which may influence responsiveness to VR-based training. Taken together, these factors indicate that the effects of VR are context-dependent and are unlikely to be fully captured by a single pooled estimate.

### Limitations

This review has several limitations. First, many of the included trials had relatively small sample sizes, and some were pilot or feasibility studies, which may have reduced statistical precision and increased the influence of random error (15, 19). Second, substantial clinical and methodological heterogeneity was present across studies, particularly in terms of participant profile, intervention content, immersion level, and training dosage. Third, several subgroup analyses were based on only a small number of studies and should therefore be interpreted cautiously. Fourth, although risk of bias was reassessed using RoB 2, methodological concerns remained in some trials, especially regarding the reporting of randomization procedures and deviations from intended interventions. Fifth, publication bias and other small-study effects could not be reliably excluded because fewer than 10 studies were available for each pooled outcome. Sixth, most studies included only short intervention periods and limited follow-up, making it difficult to determine the durability of VR-related benefits. Seventh, some pooled analyses required the aggregation of outcomes derived from instruments that were conceptually related but not fully interchangeable, which may have introduced additional measurement-related uncertainty. Eighth, the calculation of change-score standard deviations relied partly on an assumed correlation coefficient, and although such imputation is commonly used in meta-analysis, it may still affect the precision of pooled estimates. In addition, TMT-based executive/attention-related performance was informed by only one trial reporting TMT-A and TMT-B, and these two outcomes were derived from the same study population. Therefore, this finding should be considered preliminary and should not be interpreted as evidence from multiple independent trials. Finally, the accessibility, acceptability, and feasibility of different VR systems may vary across settings and populations, which may limit the generalizability of the findings.

Overall, the present review supports the view that VR-based interventions may provide cognitive support for older adults, particularly in global cognition, and may also benefit functional mobility. Evidence for TMT-based executive/attention-related performance remains preliminary and should not be interpreted as confirmation of a robust executive-function effect. Future research should prioritize larger, multicenter randomized controlled trials with better reporting quality, clearer separation of cognitive and functional outcomes, standardized intervention parameters, and longer follow-up periods. Additional studies are also needed to determine whether VR-based training can produce reliable improvements in executive/attention-related performance.

## Conclusion

In summary, this systematic review and meta-analysis suggests that VR-based interventions may improve global cognition in older adults and may also benefit functional mobility. Evidence for TMT-based executive/attention-related performance remains preliminary because it was derived from a single trial and should not be interpreted as robust evidence of executive-function improvement. The certainty of these findings is constrained by heterogeneity, limited study numbers within several analyses, and methodological limitations of the included trials. The available evidence does not yet support firm conclusions regarding the optimal intervention duration, preferred immersion level, or superiority in specific participant subgroups. VR may be considered a promising adjunctive approach for supporting selected outcomes in older adults, but stronger recommendations will require larger, more rigorously designed randomized controlled trials with standardized outcome classification and longer follow-up.

## Data Availability

The original contributions presented in the study are included in the article/supplementary material, further inquiries can be directed to the corresponding author/s.
